# Methodological Comparison between a Novel Automatic Sampling System for Gas Chromatography versus Photoacoustic Spectroscopy for Measuring Greenhouse Gas Emissions under Field Conditions

**DOI:** 10.3390/s16101638

**Published:** 2016-10-03

**Authors:** Alexander J. Schmithausen, Manfred Trimborn, Wolfgang Büscher

**Affiliations:** 1 Institute of Agricultural Engineering, University of Bonn, 53115 Bonn, Germany; m.trimborn@uni-bonn.de (M.T.); buescher@uni-bonn.de (W.B.); 2 Institute of Crop Science and Resource Conservation (INRES), University of Bonn, 53115 Bonn, Germany

**Keywords:** greenhouse gases, emissions, environmental monitoring, naturally ventilated buildings, dairy cattle, nitrous oxide, gas chromatography, photoacoustic, automated sampler

## Abstract

Trace gases such as nitrous oxide (N_2_O), methane (CH_4_), and carbon dioxide (CO_2_) are climate-related gases, and their emissions from agricultural livestock barns are not negligible. Conventional measurement systems in the field (Fourier transform infrared spectroscopy (FTIR); photoacoustic system (PAS)) are not sufficiently sensitive to N_2_O. Laser-based measurement systems are highly accurate, but they are very expensive to purchase and maintain. One cost-effective alternative is gas chromatography (GC) with electron capture detection (ECD), but this is not suitable for field applications due to radiation. Measuring samples collected automatically under field conditions in the laboratory at a subsequent time presents many challenges. This study presents a sampling designed to promote laboratory analysis of N_2_O concentrations sampled under field conditions. Analyses were carried out using PAS in the field (online system) and GC in the laboratory (offline system). Both measurement systems showed a good correlation for CH_4_ and CO_2_ concentrations. Measured N_2_O concentrations were near the detection limit for PAS. GC achieved more reliable results for N_2_O in very low concentration ranges.

## 1. Introduction

Emissions of greenhouse gases (GHGs) from agriculture livestock have global significance and potential long-term consequences. Ruminants produce climate-related gases and pollutants [1]. Measurement systems for registering air emissions provide an important overview of the source of emissions and the options for mitigating them. The main focus in agriculture is on climate-related GHGs such as carbon dioxide (CO_2_), methane (CH_4_), and nitrous oxide (N_2_O). N_2_O has a much higher CO_2_ equivalence factor (298) than CH_4_ (25) [2]. CH_4_ is mainly generated in the digestive system of ruminants and is ejected by eructation, but it also accrues during the storage of barn manure and via application to the soil [3]. N_2_O emissions are also produced by microbial nitrification and denitrification through ammonium and nitrate arising from urine and feces out of the barn [4]. N_2_O emissions are part of organic manure management; however, there is limited information about N_2_O emissions originating from animals and the barn area [5]. Data concerning naturally ventilated barns, which are the most popular design in modern intensive dairy production, is lacking.

The infrared photoacoustic system (PAS) is a common method for long-term gas measurements under field conditions. The photoacoustic multi gas analyzer (Innova 1412, LumaSense, Ballerup, Denmark) has been used in previous agricultural studies addressing gas emission. Measurements in naturally ventilated dairy barns cause a high air ventilation rate, which dilutes the trace gases. N_2_O appears in lower concentrations than other climate-related gases, resulting in a low detection rate. Uncertainties with regard to the detection limit of a photoacoustic analyzer have been described by other authors [6,7]. For PAS, there are also interferences such as water vapor. Analyses made using gas chromatography (GC) (N_2_O and CO_2_ with an electron capture detector (ECD) and CH_4_ with a flame ionization detector (FID)) are more accurate. The ECD has a ß-emitter with ^63^Ni isotope; therefore, the GC device is not portable and must be installed in a laboratory. For this reason, a system capable of taking many gas samples for long-term measurements under field conditions is needed to greatly reduce required manual sampling. 

The trace gases CH_4_ and CO_2_ can be used to evaluate both techniques [8]. Good correlations of CH_4_ and CO_2_ concentration by PAS and GC indicate whether this high-resolution system also works with N_2_O. 

Consequently, the aims of this study were to:

(a). measure the climate-related gases CO_2_, CH_4_, and N_2_O with two different measurement systems (online and offline) under field conditions;
(b). validate the measurements of online (infrared photoacoustic) and offline (gas chromatography) techniques to improve the best method of N_2_O detection.

## 2. Materials and Methods 

### 2.1. Analysis of Gas Concentrations

#### 2.1.1. Photoacoustic Technique

The concentrations of CO_2_, CH_4_, and N_2_O gases in the collected air samples were measured using a 1412 photoacoustic multi gas analyzer and a 1303 multiplexer (to switch between the sections) (LumaSense Technologies SA, Ballerup, Denmark). Table 1 shows the detection limits calculated by threefold standard deviation of the calibration chart after the last calibration by the manufacturer. Additional settings of the measurement software are the compensation of water and cross interference.

#### 2.1.2. Chromatographic Technique

Samples were analyzed using a gas chromatograph from SRI Instruments (8610 C, SRI Instruments, Torrance, CA, USA). N_2_O and CO_2_ were analyzed using an electron capture detector (ECD) (Table 1). For CH_4_, a flame ionization detector (FID) was used in accordance with [9,10]. The detection limits for GC were calculated from the threefold standard deviation of multiple analyses of standard gases with ambient concentrations of CO_2_, CH_4_, and N_2_O (Table 1).

### 2.2. Experimental Setup

Gas Sampling System

Trace gases emitted from a naturally ventilated dairy barn (68 m × 34 m) were measured to calculate the emission rates. To calculate emission rates, a quantitative measurement technique to analyze the respective trace gases was needed. The air exchange rate was also important to calculate the emission rate. Equation (1) shows the calculation for the emission rate (${\dot{m}}_{x}$) based on volume flow rate ($\dot{V}$) and the gas concentration inside ($C_{x_{in}}$) and outside ($C_{x_{out}}$) the barn:

(1) $${\dot{m}}_{x}=\dot{V}\times \left(C_{x_{in}}-C_{x_{out}}\right).$$

**Figure 1:**
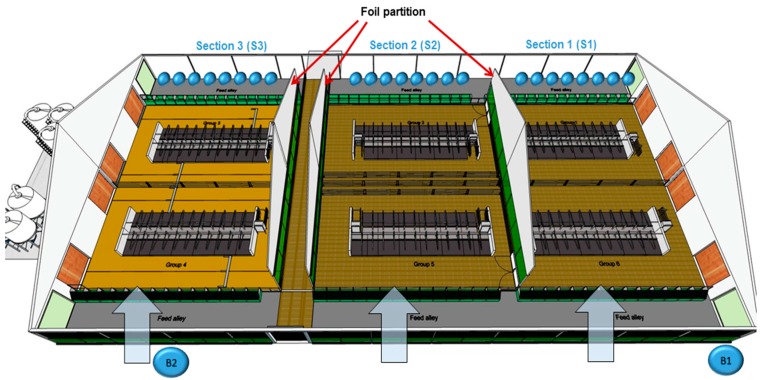
Top view of the barn (68 m × 34 m) showing the three different sections with the sampling points at the lee side of the barn (S1, S2, S3) and the background sampling points (B1, B2).

**Figure 2:**
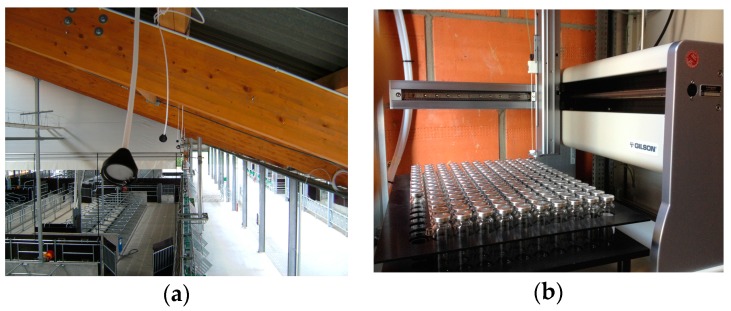
(**a**) The sampling point at the leeward side of the barn to measure gas concentrations. (**b**) The offline system (Gilson, GX-271 sampler) with evacuated glass vials.

This study focuses on the quantitative acquisition of trace gas concentrations. Therefore, the barn building was divided into three separate air sections. Section 1 and Section 2 (S1, S2) were equipped with a slatted floor, while Section 3 (S3) was equipped with a solid floor system. Two sampling points were installed outside the barn to measure gas concentrations in the background air (B1, B2). The background air was measured at the windward side of the barn, while the gas concentrations in the outgoing air of the equally divided sections were measured at the leeward side (Figure 1). Eight sampling points were merged to form one aggregate sample for each section. The sampling points were located 4 m above the feed alley and were equipped with filter orifices to protect them from dust (Figure 2a). 

**Figure 3:**
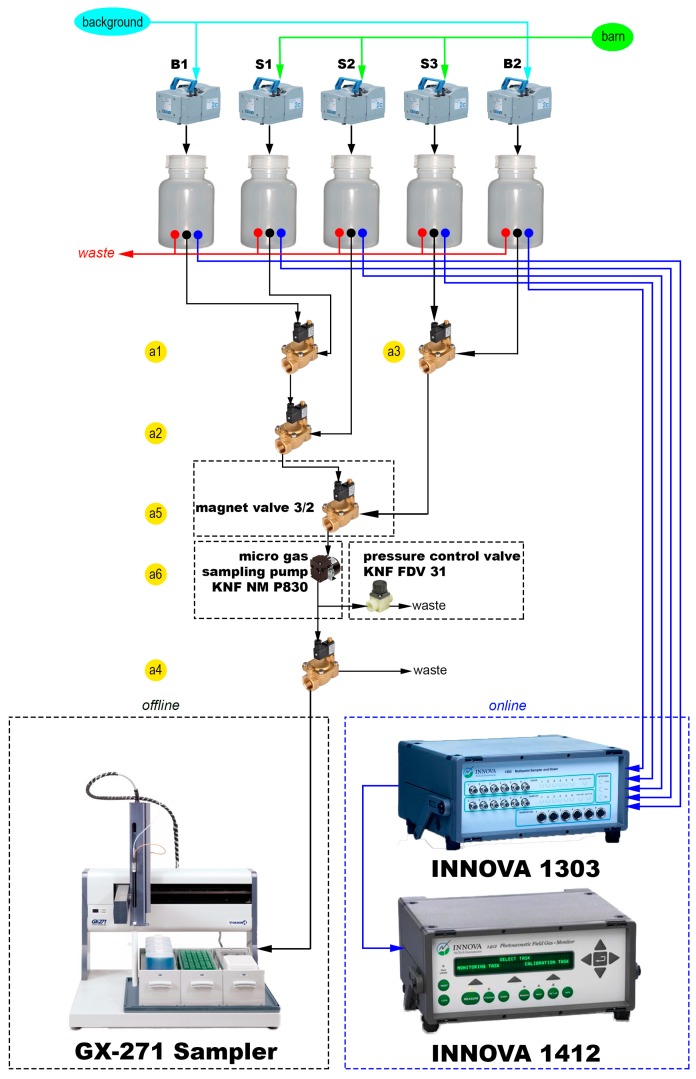
Schematic diagram of the experimental setup. Online: infrared photoacoustic measurement in real time; offline: samples analyzed using gas chromatography in the laboratory.

The air samples from the dairy barn were pumped continuously using vacuum pumps (ME 2C, Vacuubrand GmbH + Co. KG, Wertheim, Germany) with a suction capacity of 33 L/min through 100-m PTFE (polytetrafluoroethylene) tubes into five separate sampling bottles (600 mL, Figure 3). Samples were taken from the online and offline systems independently. The online system worked with the multiport INNOVA 1303 made by the manufacturer of the photoacoustic analyzer (Figure 3). The online system had an interval of 60 s for each sample and thus required 5 min for the complete sample cycle.

**Figure 4:**
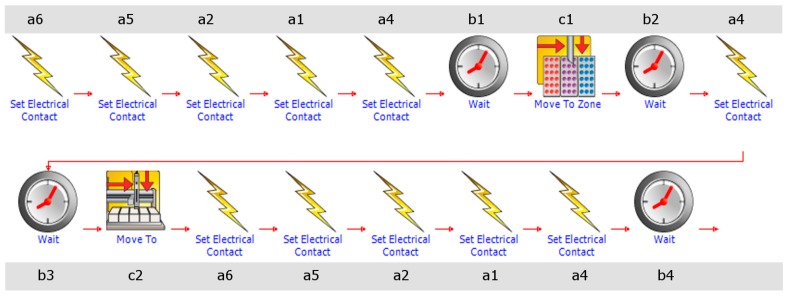
A schematic circuit diagram of the offline system (Gilson Sampler GX-271; Software: TRILUTION LH 3.0, Gilson, Inc.); (a: electrical circuit of the magnet valves and the sampling pump; b: wait times between the processes; c: order from the software to move the arm of the GX-271 in the *x*-, *y*-, or *z*-direction).

The offline system consisted of magnetic valve switches and a liquid handler system (Liquid Handler GX-271, Gilson, Middleton, WI, USA). This system fills up gas samples into evacuated headspace vials (20 mL) (Figure 3). The system had a capacity for 165 vials (specially customized). Based on the XYZ-technique, it is a compact and space-saving system for measuring under field conditions. The GX-271 sampler is a commercial system controlled by manufacturer software (TRILUTION LH 3.0, Gilson, Inc.). The liquid handler was programmed to prick the evacuated vials after a default time interval. For the offline system, this interval was also 60 s for five samples. One sampling interval was implemented every three hours. The headspace vials were evacuated in the laboratory, so the GX-271 sampler only had to prick the needle into the rubber septum of the vial (Figure 2b). All tubes from the offline system were continuously purged using a micro gas sampling pump (KNF NM P830, KNF Neuberger GmbH, Freiburg im Breisgau, Germany). A pressure control valve was installed (KNF FDV 31, KNF Neuberger GmbH) between the sampling pump and the waste valve to bridge periods of overpressure (Figure 3). Both the online system (serial port, COM 1) and offline system (RJ45 interface) were controlled by the same computer and through remote servicing via the internet. To operate the electrical contacts, the Gilson 506C System Interface (serial port, COM 2) was used. Figure 4 shows the circuit diagram for the 3/2-way magnetic valves of the GX-271 sampler. The magnetic valves were switched for each sampling point (sampling bottles; Figure 3). This is similar to the multiport INNOVA 1303 online system. Figure 4 provides an example of sampling in Section 1. First, the sampling pump was supplied with electricity (a6); second, the 3/2-way magnetic valves were switched (a5, a2, a1) to reach the correct sampling bottle. Magnetic valve a4 was also opened to flush the entire tube system with the selected sampling air. The wait instruction (b1) was the same as the time needed to flush the tube system (20 s). The instruction “move to zone” (c1) made the arm of the GX-271 move to the defined glass vial and prick in. The glass vial was filled over a period of 20 s (b2). Subsequently, magnetic valve a4 was switched for 5 s (b3) to release the overpressure within the vial. Immediately afterwards, the needle was pulled out of the vial (c2), and all electrical contacts were set to off (a6, a5, a2, a1, a4) until the next measurement was started (b4).

### 2.3. Data Measuring and Evaluation

The GHGs were measured via the PAS (online system) from the same sampling point approximately every five minutes. The offline system measured every three hours at the same interval as the online system. To make the data coherent, the log file data from both systems were analyzed and compared with regard to time. Only time differences less than 30 s were included in the evaluation. Offline system sampling every three hours afforded the opportunity to take eight samples per day (capacity of 165 vials and five sampling points) for four days of sampling. This was needed for later long-term measurements of N_2_O in naturally ventilated barns. Data for this study were collected in October 2015 over a representative period of three days from four sampling points (B1, S1, S2, B2). Four samples were taken daily at three-hour intervals (eight samples per day). The 96 samples provided a basis for comparing both systems. 

### 2.4. Statistical Methods

The statistical software SPSS Statistics 23.0 (IBM, New York, NY, USA, 2015) was used to compare the measured concentrations. The data was checked relative to the normal distribution (Kolmogorow–Smirnov test). A Student’s t-test and the Wilcoxon test were used to identify significant differences between the detection techniques. Statistical significance was achieved on reaching a least significant difference (LSD) value of 95% confidence (*p* < 0.05). Regression analysis uses the coefficient of determination (*R*^2^) to calculate significance levels.

We hypothesized that the GC technique is more accurate for measuring low concentrations of N_2_O, whereas the PAS system is easier to handle and has shorter response times for measuring gas concentrations.

## 3. Results

### 3.1. Data Evaluation

**Figure 5:**
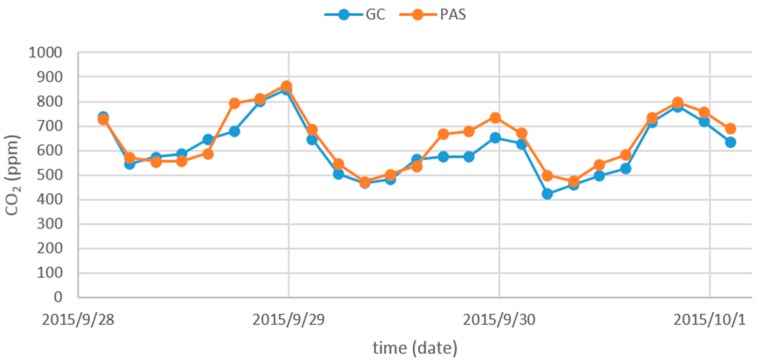
Absolute concentration of CO_2_ measured by GC and PAS.

Gas concentrations with a wide range of variability are required to compare measuring systems. In this study, these variability of the gas concentrations were caused by different sampling points and the switching between background sample and exhaust air of the barn. The large variability in gas concentrations was caused by changes in the rate of air exchange as a result of wind velocity and the animal activity. Figure 5 shows the typical diurnal variations of CO_2_ concentration over the measurement period in the naturally ventilated barn. The CO_2_ concentration in Section 1 allows a comparative overview of PAS and GC. This is the natural situation and not an artefact of the measurement technique. 

### 3.2. Results for the Sampled Gases

**Figure 6:**
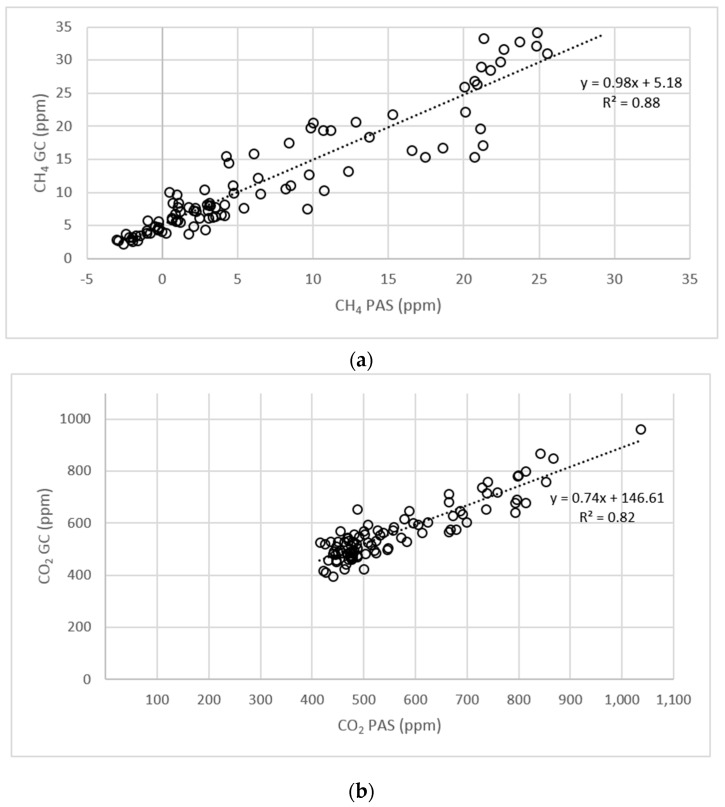

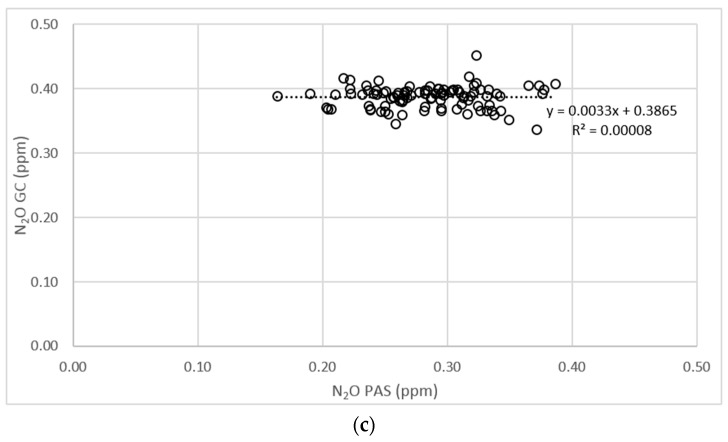
Correlation among CH_4_ (**a**), CO_2_ (**b**), and N_2_O (**c**) concentrations detected using photoacoustic spectroscopy (PAS) and gas chromatography (GC).

Table 2 presents the average concentrations of CH_4_, CO_2_, and N_2_O gases from a total of 96 values (*n* = 96). For PAS, the concentration ranges were 3.03–29.15 ppm, 414.9–1035.3 ppm, and 0.16–0.39 ppm for CH_4_, CO_2_, and N_2_O, respectively. For GC, the concentration ranges were 2.26–35.78 ppm, 397.10–960.82 ppm, and 0.34–0.49 ppm for CH_4_, CO_2_, and N_2_O, respectively. The mean concentration determined by PAS showed a zero-point drift, especially for CH_4_ and N_2_O (Table 2; Figure 6). N_2_O concentration (measured by PAS) was obviously lower than the concentration in ambient air (0.35–0.36 ppm).

### 3.3. Comparison of Measurements Obtained with PAS and GC

Figure 6 shows the concentrations of CH_4_, CO_2_, and N_2_O in air samples collected via PAS and GC. CH_4_ and CO_2_ concentrations showed good correlation between PAS and GC (*R*^2^ = 0.88 and 0.82; *p* < 0.01). The N_2_O concentration measured by PAS and GC measurement systems showed no correlation (*R*^2^ = 0.00008; *p* > 0.05). In addition, CH_4_ measurements taken via PAS showed a zero-point drift, but there was a near 1:1 relation (*R*^2^ = 0.88) between the CH_4_ concentrations measured using PAS and GC (Figure 6a). In the lower dispersion of the values, the concentration range of CO_2_ was more pronounced (*R*^2^ = 0.82) compared with CH_4_ (Figure 6b). Data collected using PAS showed a higher dispersion than those collected using GC. The majority of N_2_O values using PAS were still below the environmental (background) concentration of 0.35 ppm in a range of 0.16–0.39 (Figure 6c). This was attributed to the zero-point drift of the PAS.

## 4. Discussion

Different gas concentrations were measured using PAS and GC. The correlation for CH_4_ (*R*^2^ = 0.88) between PAS and GC was relatively high, indicating that both techniques are suitable for CH_4_ analysis. For this reason, the CH_4_ concentration was used to test the functionality of the offline sampling system. The concentration of CO_2_ was also well correlated (*R*^2^ = 0.82), similar to [11]. This is because the variance of CH_4_ and CO_2_ concentrations were large enough (Table 2). CO_2_ concentration were at a range of 600 ppm (Figure 6b). This is clearly higher than the detection limit of the CO_2_ concentration using PAS (12 ppm) and GC (50 ppm). Such an approach results in a good correlation between PAS and GC. For N_2_O concentration, PAS and GC did not correlate (*R*^2^ = 0.00008). The mean N_2_O measured via PAS (0.284 ppm) was seven times higher than the PAS detection limit. However, despite all settings of the PAS software (water and cross interference), detecting interferences between gases cannot be excluded. GC analysis of N_2_O concentration in the laboratory was more precise, because the GC detection limit is 0.01 ppm (Table 1). The variability of N_2_O concentration by PAS could suggest that these differences in concentration are caused by emission rates from the barn, whereas the values by GC show a low variability and are close to the ambient air concentration (Figure 6c). This is obviously a misinterpretation of emission rates by PAS. Similar results for N_2_O measurements with PAS have been confirmed in the literature [6,7]. Jungbluth [12] observed more variation in N_2_O compared with CH_4_ due to low N_2_O concentrations. Therefore, the offline system described here is an attractive alternative technique for collecting samples under field conditions for subsequent laboratory analysis. Overall, the average CH_4_ concentration measured using PAS was 42% lower than that measured using GC. This underestimation of CH_4_ by PAS has also been described by [12,13]. Another point to consider when using PAS is the humidity (such as condensed water) in the tube system. Cortus [9] mentioned the influence of humidity on gas concentrations lower than 20 ppm. For this reason, the last 15 m of the tube system is heated to minimize the influence of temperature and condensation [14].

Because of the zero-point drift, especially for CH_4_, frequent calibration of the measurement system is recommended. There were more deviations (SD) in the lower concentration ranges than the higher ones. A drift of 0.5 ppm (SD) below a concentration of 5 ppm represents an average relative error of 500%. In the lower concentration ranges, the dispersion of CO_2_ values was more pronounced (*R*^2^ = 0.82) than CH_4_ values (*R*^2^ = 0.88) (Figure 6b). This is due to the weak analysis of CO_2_ by GC-ECD. Other GC detectors, such as a methanizer combined with a FID, are more sensitive to CO_2_ but were not available in this study.

Future studies should collect more measurements, and PAS should be calibrated by time. In addition, calibration gases should be used to test the accuracy of the technique [15,16]. It is also important to evaluate the analyzers selected for GHG measurements and conduct tests to ensure the chosen analyzers consider the different variations [17]. GC is a known system that is used to classify and validate other measurement techniques such as PAS and cavity ring-down spectroscopy [18]. Partly differences between PAS and GC can be caused by interference of diverse reasons. Therefore, it is important to use additional settings like cross and water compensation when measuring with PAS. Furthermore, additional accurate laboratory measurements with test gases are necessary. 

## 5. Conclusions/Outlook

This study has demonstrated that many N_2_O samples can be taken under field conditions, which are analyzed in laboratory. These values from laboratory are more accurate and can therefore be used to adjust the values measured by PAS. Both PAS and GC yielded comparable CH_4_ concentrations. However, N_2_O results gathered with GC were more precise than those gathered with PAS. The PAS detection of N_2_O is only 25% of GC. The GC is calibrated by threefold standard deviation with CO_2_, CH_4_, and N_2_O as standard gases. Qualitative measurements to generate emission calculation are important for relevant environmental monitoring. 

In future studies with the same offline GC system, it is conceivable that sulfur hexafluoride (SF_6_) can be sampled as a tracer within the barn to calculate the ventilation rate of the building via the tracer gas technique applied by [19,20]. Concentrations of SF_6_ can be measured by GC and ECD down to the parts per thousand (ppt) range. 

## Figures and Tables

**Table 1:** Comparison of the gas analyzers used for detecting CO_2_, CH_4_, and N_2_O with the infrared photoacoustic system (PAS) and gas chromatography (GC).

Division	PAS	GC
manufacturer	LumaSense Technologies, Ballerup, Denmark	SRI Instruments, Torrance, CA, USA
analyzer model	INNOVA 1412	8610c
detection method	infrared photoacoustic	ECD & FID
target gas/detection limit	CO_2_ 12 ppm	CO_2_ 50 ppm
	CH_4_ 0.55 ppm	CH_4_ 0.08 ppm
	N_2_O 0.04 ppm	N_2_O 0.01 ppm
	NH_3_ 0.3 ppm	NH_3_ incapable
response time	1 min (on site)	8 min (in laboratory)

**Table 2:** Analysis of gas concentrations using the PAS and GC techniques.

Method of Analysis	Measured Gases	Measured Concentration (ppm)	
	*n* = 96	Mean	SEM
PAS	CH_4_	6.83	0.87
	CO_2_	559.63	13.34
	N_2_O	0.284	0.005
GC	CH_4_	11.87	0.91
	CO_2_	563.21	10.96
	N_2_O	0.389	0.002
